# Framework for planning and monitoring active TB case finding interventions to meet the global targets in the COVID-19 era and beyond: South-East Asia perspective

**DOI:** 10.1371/journal.pgph.0000073

**Published:** 2021-11-23

**Authors:** Alka Aggarwal Singh, Jacob Creswell, Vineet Bhatia

**Affiliations:** 1 Independent Senior Public Health Consultant, New Delhi, India; 2 Stop TB Partnership, Geneva, Switzerland; 3 South-East Asia Regional Office, World Health Organization, New Delhi, India; Boston University, UNITED STATES

## Abstract

There was an estimated 20–40% decline in tuberculosis (TB) case detection in the South-East Asia Region (SEA Region) during 2020 due to COVID-19 outbreak. This is over and above a million people with TB who were missed each year, prior to the pandemic. Active case finding (ACF) for TB has been gaining considerable interest and investment in the SEA Region and will be even more essential for finding people with TB missed due to the COVID-19 pandemic. Many countries in the Region have incorporated ACF activities into national strategic plans and are conducting large scale activities with varying results. ACF can reach people with TB earlier than routine approaches, can lead to increases in the numbers of people diagnosed, and is often needed for certain key populations who face stigma, social, and economic barriers. However, ACF is not a one size fits all approach, and has higher costs than routine care. So, planning interventions in consultation with relevant stakeholders including the affected communities is critical. Furthermore, continuous monitoring during the intervention and after completion is crucial as national TB programmes review progress and decide on the effective utilization of limited resources. Planning and monitoring become more relevant in the COVID-19 era because of constraints posed by resource diversion towards pandemic control. Here, we summarize different aspects of planning and monitoring of ACF approaches to inform national TB programmes and partners based on experiences in the SEA Region, as programmes look to reach those who are missed and catch-up on progress towards ending TB.

## Background

The South-East Asia Region (SEA Region) of the World Health Organization (WHO) has the highest tuberculosis (TB) burden among all six WHO Regions. New TB case notifications in the SEA Region had improved by more than 30% between 2015 and 2019 [1], but the decline in incidence was estimated at less than 3% annually. Close to a million people with TB are missed every year in SEA Region [1] and to meet the UN high level meeting targets for TB, the region has to detect and notify more than 18 million people in the 2018–2022 period, almost 50% of the global 40 million target [2]. The gaps in case detection were exacerbated during the COVID-19 outbreak. Preliminary data released by WHO show declines in TB case notification ranging from 20% to 40% in 2020 due to COVID-19 related restrictions and strains on the health system imposed by the COVID-19 response [3]. If catch-up activities, including active case finding (ACF) are not urgently planned and implemented, then extrapolating from global modelling predicting excess mortality [4], the SEA REGION could witness more than 700,000 additional deaths due to TB over next 3–5 years.

Recently published WHO guidance recommends screening for TB for contacts of people with TB, people living with HIV and several key populations including miners, prisoners, migrants, and indigenous populations [5]. With the help of a tool developed by Stop TB Partnership various countries in SEA REGION including Bangladesh, India and Indonesia have employed ACF to engage various key populations (KP), those that experience a high epidemiological impact from one of the diseases, combined with reduced access to services and/or being criminalized or otherwise marginalized [6]. ACF activities are part of many National Strategic Plans (NSP)s of SEA REGION countries [7, 8]. ACF interventions are generally more resource intensive than passive case finding [9]. However, not all ACF interventions are impactful [10] and deficiencies in reporting make it difficult to properly evaluate many of them [11]. Therefore, careful planning and monitoring of ACF activities is required to improve yield, efficiency, and mid-course correction when needed [12]. In mid-2021 WHO SEA Regional office published “Optimizing active case finding for tuberculosis: Implementation lessons from South-East Asia” [13].

Here, we present a summary of the findings on ACF for TB and offer a planning and monitoring framework of to improve the performance and effectiveness of ACF activities.

## Experience of ACF in the region

The review of ACF projects showed increases in overall case notifications across several projects in SEAR and beyond. Potential benefits of ACF include reducing access barriers especially among key populations (KPs), reducing the costs of diagnosis and cure of TB. Many recent studies have focused on documenting the results of contact investigation which have yields as high as 5% in southern India [14]. Other ACF interventions have focused on communities and groups with limited access to care where prevalence has been very high among some populations like tribal groups in India where TB prevalence identified by smear microscopy was documented at 1.5% [15]. High rates of TB were also documented through outreach efforts to KPs in the Philippines [16]. However, despite increasing case notification in intervention areas, the impact on national notifications was variable, likely due to limited coverage and a shift in diagnoses from passive case finding to ACF. Some ACF interventions were not successful in increasing case notifications, e.g. a project in Indonesia screened 5,100 individuals in selected neighbourhoods yet no individual with smear positive TB was diagnosed [10]. Publication bias likely limits the reporting of many less successful ACF interventions. Possible explanations for the lack of detection included that ACF activities were of inadequate duration, used poor sensitivity screening algorithms, or because the scale of the intervention was too small to make a significant contribution. Additional challenges for conducting ACF include access to good data, especially at a community level, higher costs, and the selection of populations or groups to prioritize. Appropriate planning and systematic monitoring could help improve outcomes of ACF interventions and improve cost-efficiency.

### Challenges for ACF in the region

Major challenges for the community that decreased their participation in ACF activities were stigma, a lack of awareness about TB, lack of family support, fear of a loss of wages, transportation costs, and a preference for private health care because of overcrowded public hospitals [14, 17–20]. Challenges from the health providers’ perspective were a lack of motivation due to a target-oriented approach, insufficient incentives, inappropriate timing of ACF, a lack of cooperation from other health staff, and diversion from their usual work to do home visits, community screening or other activities outside health facilities [21, 22]. The challenges noted for the technical experts were selection of population groups, frequency of screening, selection of screening and diagnostic algorithms against the feasibility of implementation in the target population, costs and the yield of algorithms [23, 24]. The managerial challenges for implementing ACF activities were coordination of activities in large populations, staff management for house-to-house visits, camps in remote areas, mobilization of screening and diagnostic equipment, staff training and supervision, sample transfer from field to laboratories/mobile vans, and quality assurance of testing [10, 15, 25]

**Fig 1:**
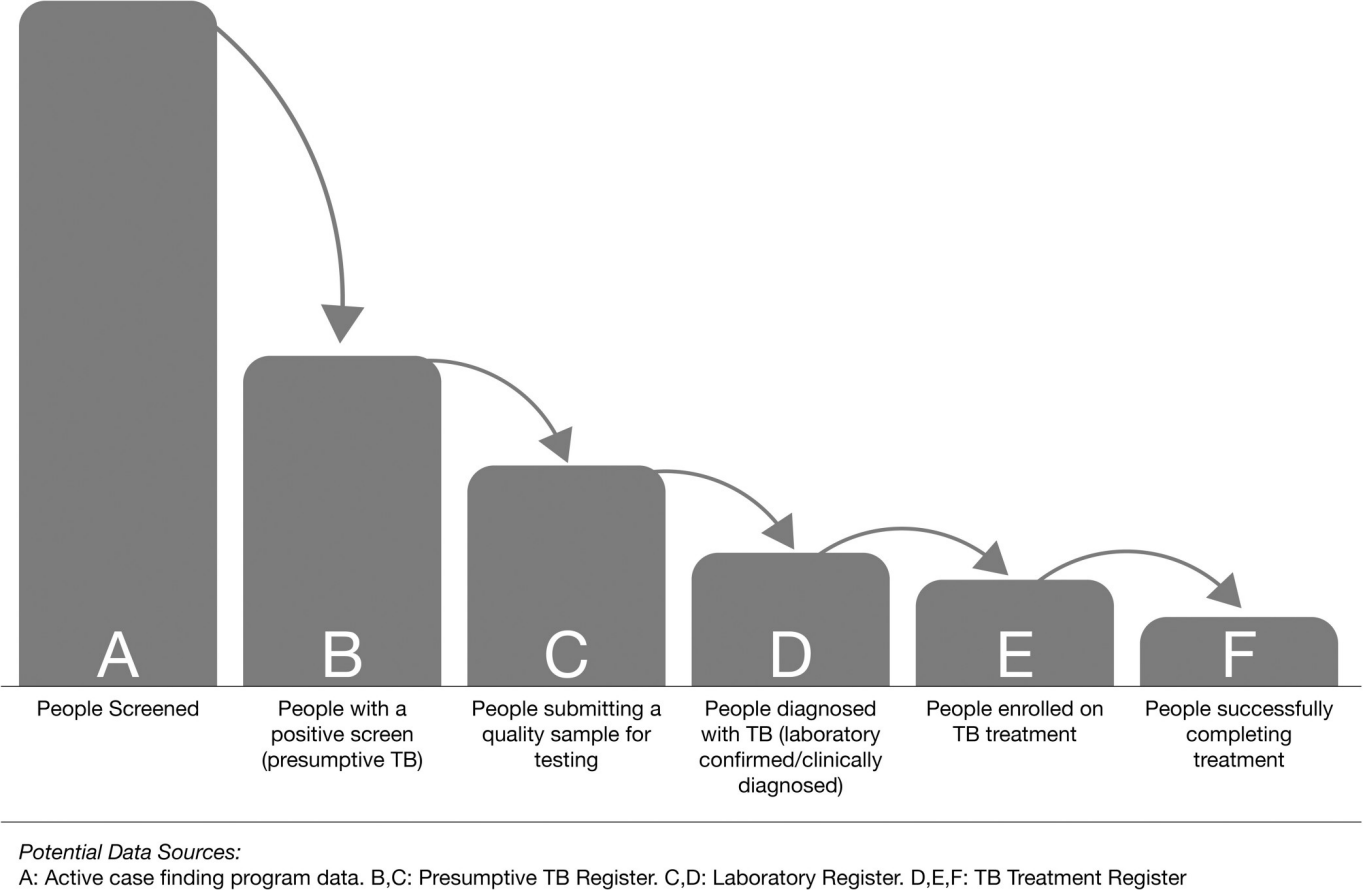
The TB screening cascade.

ACF generally involves a series of steps that include engaging people to participate, screening them for presumptive TB using symptoms or chest X-ray (CXR), collecting sputum, testing the sputum and linking people with their results and treatment. This process is often referred to as the TB screening cascade (Fig 1). The figure provided in this manuscript is representative of field situation but is not based on an actual data set. Readers can adopt this figure to their context for further analysis and target setting in specific populations.

Sub-optimal implementation of a technically sound strategy for ACF can lead to a loss of people at various stages of the cascade of screening, diagnosis and treatment, thereby resulting in decreased yield and cost-effectiveness [14, 20]. Additionally, with the recent COVID-19 pandemic, restrictions on movement and social gatherings have limited the implementation of community outreach activities which are integral to many ACF efforts. Given the respiratory nature of both disease and similar presentations, many governments and WHO recommend screening people for both diseases, but this bi-directional screening has been inconsistently applied for many reasons including lack of comprehensive guidelines, adequate personal protective equipment for staff, access to common laboratory platforms, stigma and lack of care seeking behaviour [26].

### Good practices in the region

Community participation was increased by use of media for dissemination of messages [27, 28], incentives and house to house visits [10]. The quality of screening was improved by training of volunteers, incentives for volunteers, symptom-checking with individual household members instead of head of the household improved the quality of screening [14]. Support measure such as sputum transportation, on-site testing [29, 30] travel with a volunteer, and travel compensation decreased the drop-out for testing [30]. Engagement of community volunteers [14, 20] and the use of mobile health technology to track screening decreased the drop-out from initiation of treatment [14, 16]. Quality of diagnosis was maintained by use of rapid molecular testing, quality assurance of sputum microscopy, and use of artificial intelligence software to quickly and efficiently read high volumes of CXR [31, 32]. As ACF campaigns usually include large increases in laboratory testing and people treated, the engagement of non-governmental organizations (NGOs) [16] and private sector [32, 33] prevented the over-burdening of local health facilities and the diversion of workers. Other activities that improved the cost-efficiency and improved monitoring, linkage to care and reporting included the use of digital system for recording patient data and to coordinate between screening, diagnosis and treatment initiation, and integrating ACF activities for TB with other health programmes to optimize resources. In addition, combining ACF with providing TB preventive treatment (TPT) to prevent future disease is encouraged since many of the populations eligible for TPT are contacts of an individual with active TB disease [34].

## The suggested planning framework for ACF

The planning framework for ACF (Table 1) presents multiple steps to enable feedback and course corrections when implementing an intervention. In the past, ACF has often been conducted by NGO partners with better access to the community, but the integration within a national response has lacked. This lack of coordination can cause inefficiencies in improving case detection. The framework is aligned with the recent WHO SEARO publications and global guidelines and other global recommendations for ACF [13, 35].

**Table 1:** Planning activities for ACF.

Step Number	Theme	Consideration
1	Plan timing	NSP development following a programme review/joint monitoring mission.
		With COVID-19 impacting case finding activities, this needs to be done on immediate, ad-hoc basis in several countries.
2	Engaging the stakeholders	implementers with ACF experience;
		NGOs and civil society organizations engaged in COVID-19/TB activities;
		social scientists with knowledge of key populations, representatives of these groups, civil society, local leaders,
		health care providers in public and private sector, and medical colleges
		TB experts including paediatric TB experts,
		Sub-national programme managers of respective area
3	Desk review	Published and unpublished ACF studies in the country, and similar setting in other countries.
		prevalence survey data, census data, TB surveillance data (national data as well as state or district level);
		CRG assessment tool [6] and spatial mapping approaches can be used [36].
4	Defining the objective of ACF	Any or a combination of–to increase TB notification (additional case yield), to detect cases early, to overcome barriers as human rights of KPs, to address out of pocket costs for individuals with TB;
5	Selection of populations for ACF	Listing of all KPs in the country with their size, description of their relevant characteristics (geographic area, access to services, etc.).
		Mapping of areas with high population density and a significant fall in case finding for the COVID-19 catch-up planning.
		For long term purpose, baseline prevalence of TB in the proposed population group/s is important.
6	Selection of a screening and diagnostic algorithm	Size of population for ACF, estimated TB burden, available infrastructure and partners in the vicinity of the population, costs of screening and diagnostic tests.
		Human resource expertise, cost and availablity;
		Systems for efficient and quality assured services;
		Frequency of ACF rounds in the population groups (quarterly, semi-annual, annual, etc), potential use of rapid diagnostic tests for COVID-19 and other disease for people with chest symptoms/fever
7	Early treatment initiation	Areas facing restricted access to health services. During COVID-19 outbreak, this might happen through tele -consultation.
8	Inclusion of TPT	TB preventative treatment among contacts who are not found to have active TB disease.
9	Training and protection of workers	PPE kits and training of outreach workers, during and beyond the COVID-19 pandemic.
10	Cross-referral for synergies with other programmes	While COVID-19 screening may need immediate attention, for long term purposes, cross referral with diabetes management programmes, tobacco cessation and nutrition rehabilitation will be important
11	Engaging the local community and civil society	Community engagement at national level with the aim of—partnership at local levels to increase participation and prevent dropouts;
		community-led monitoring, consultations with the community and sharing of lessons learnt would build partnership.
		Community empowerment for appropriate diagnosis and care (for COVID-19, TB and other diseases, as the case may be) of those identified by the ACF activities.
12	Logistics	Map additional local capacity for implementing ACF (for example, for house visits, sputum transportation, follow up with those found to have presumptive TB, and with those found to have the disease). These could be partners from civil society and private sector.
13	Planning for M&E	this includes focal person at the national level, staffing for M&E at the national and local level, consent, community level monitors, equipment, dissemination of results, etc.
		Selection of indicators and their measurement, training for M&E, focus group discussions, if planned.
		Analyse results against the objectives including additional notification, costs saved for individuals, and costs for the NTP;
		Document the lessons learnt and disseminate with all relevant stakeholders.
		Investment in operational research to improve the next rounds.

### Step 1—Timing of planning for ACF activities

The planning for ACF should be mainstreamed into the NSP and reviewed and evaluated with each iteration of the NSP. The ACF review be incorporated in a general TB programme review to elucidate challenges for improving yield and performance, and make recommendations to improve outreach. This enables the stakeholders to contextualize ACF activities within the overall program to end TB. It also helps ensure the various partners working in the TB response create efficiencies rather than conducting overlapping or contradictory interventions. While planning for ACF during the COCID-19 pandemic, when countries are striving to catch-up on case detection that declined due to lockdowns, urgent planning before the end of current NSP cycle is needed.

### Steps 2–4—Engaging the stakeholders, desk review and defining the objective

In the current scenario of an ongoing pandemic when the health system is under a huge strain, mapping of traditional stakeholders, civil society, and identifying potential new players become more important. A thorough review of domestic ACF experiences is crucial for all stakeholders to understand the gaps and strengths of earlier approaches and their relevance in the current context. An assessment of gaps in implementation and performance of the TB programme, specifically those aggravated by COVID-19 pandemic are critical. Careful analysis using TB data but also other sources including demographic, geographic and temporal data can be used to identify gaps in the current system [37, 38] The identification and size estimation of KPs, often left behind by routine services, can be conducted [39]. Modelling studies may help determine the extent of additional case finding required to mitigate the impact of reduced case finding due to COVID-19 pandemic. All stakeholders should understand and own the objectives of ACF activities that can be achieved in a particular time period given the available financial and human resources. A clear statement of objectives of the ACF activities including measures such as increased detection, early detection and/or reduced patient costs will be helpful at the initial planning stage.

### Step 5 —*Selection of population groups*

Selecting the populations for ACF can be done by modelling but the initial choice should be guided by the prevalence of TB in the group, target population size, and current access to care with an immediate focus on vulnerable populations that faced maximum disruption of services due to COVID-19 pandemic. Depending on the objectives set for the intervention, calculations of potential TB yield could guide the selection process. For example if the objective of ACF is to increase the TB notification in a region, a small population group with high prevalence of TB such as household contacts will be efficient but may not contribute as much to the total yield as much as a bigger population with slightly lower prevalence such as the rural poor.

### Step 6 —*Selection of algorithms*

**Fig 2:**
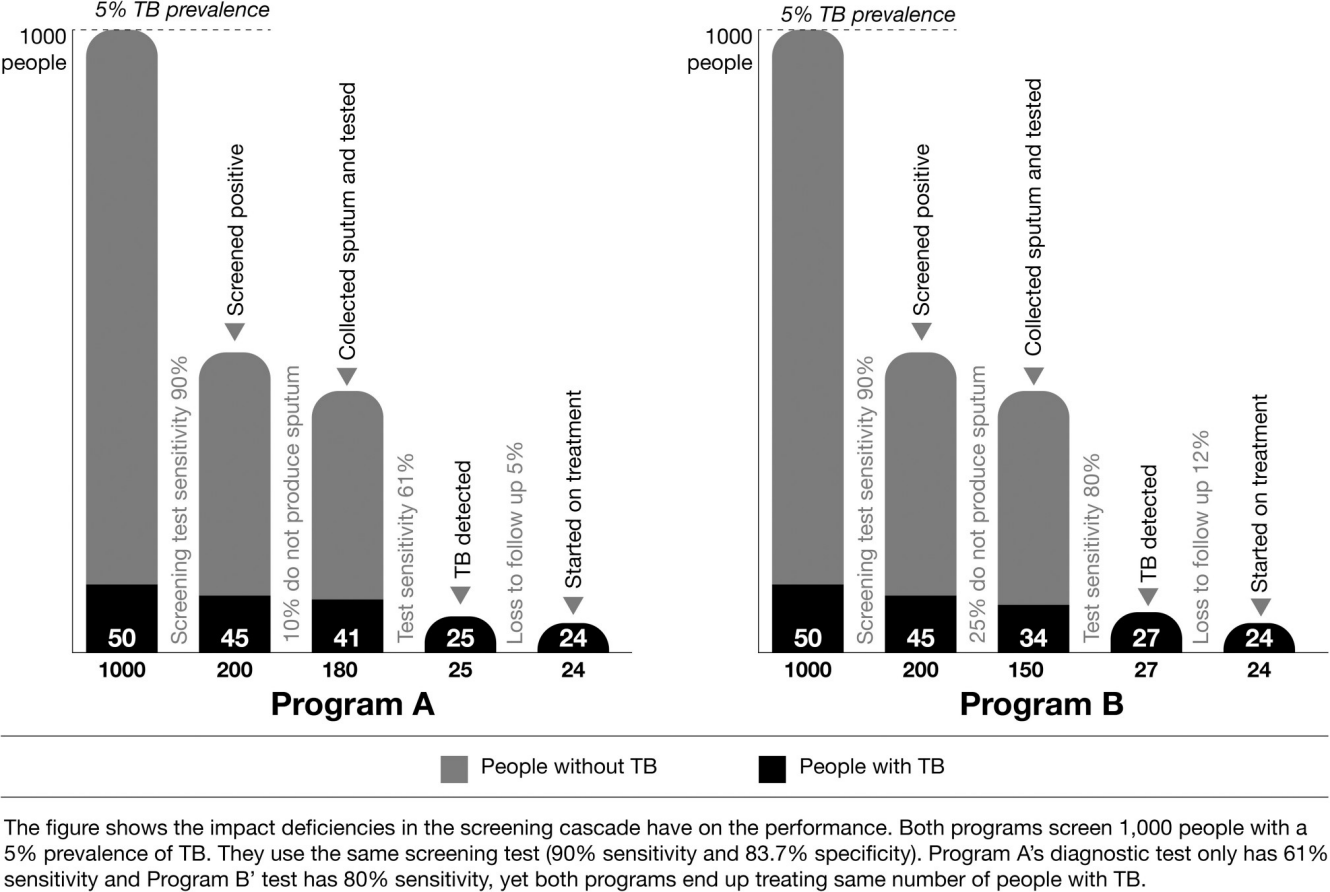
The impact of active case finding operational performance on TB diagnosis and treatment.

The yield of TB cases from ACF activities will also be determined by the screening and diagnostic algorithm used supported by a strong implementation plan. The choice of algorithm will be balanced between sensitivity, cost and the quality/feasibility of implementation. For example, if an algorithm with high sensitivity is not implemented well and there is significant leakage along the cascade of screening to treatment completion then it will not be as effective as an algorithm with lower sensitivity and specificity which does not lose people along the cascade as shown in Fig 2.

The importance of dropouts along the screening cascade (presumptive to testing, and pre-treatment loss to follow up) has been highlighted in several ACF publications as a major limitation, but one that can be addressed [15]. Algorithms should ensure accuracy, feasibility and cost-effectiveness and avoid unnecessary treatment from false-positive results. Algorithms using rapid molecular diagnostics and CXR are more sensitive and generally recommended as first choice, but the diagnostic costs will be high [40]. Verbal symptoms screening and diagnosis using sputum microscopy are cheaper with only the human resources as the significant costs but may miss a large proportion of people with TB and lead to more false positive and false negative results [41]. The selection of algorithms should also include consideration for bidirectional screening for COVID-19 screening with rapid tests.

### Step 7—*Ensuring early treatment initiation*

Finding people with TB through ACF activities will only provide a decrease in transmission if people with TB are identified early, initiated on treatment promptly, and are supported to successfully complete treatment. Leakage in this pathway of diagnosis, initiation on treatment and treatment completion must be pre-empted during the planning phase and managed. While pre-treatment loss to follow-up has been identified as an issue in passive case finding interventions [42], it is an important issue for ACF where referrals and linkage to care can be more difficult specifically if it is not a single point of service provision [16, 43]. In the planning stages, addressing the potential areas of dropout and strengthening those linkages can improve the chances of treatment success.

### Step 8—*Including TB preventive treatment*

While planning for ACF, it is also essential to plan for TPT for those eligible. The provision of TPT is recommended by WHO to vulnerable groups such as children, people living with HIV (PLHIV) and household contacts of people with TB [5]. As ACF will often screen at-risk groups like contacts of TB patients and PLHIV for TB disease, they can also be considered for TPT. Including TPT will not only help in achieving the United Nations High Level Meeting on TB targets [2] but is part of a comprehensive strategy to decrease TB incidence.

### Step 9—*Protecting the health workers*

During the COVID-19 pandemic the importance of infection prevention and control (IPC), specifically the use of proper personal protective equipment (PPE) in controlling airborne infections has received significant attention. IPC measures, specifically the use of PPE kits for health personal during ACF is critical, as both the diseases are airborne. There will be instances when symptoms are suggestive of both COVID-19 and TB or at least they cannot be easily distinguished. Protecting outreach workers from TB and COVID-19 infection must be costed and considered in the planning stages.

### Step 10—*Synergies with other programmes*

Just as the COVID-19 pandemic has created opportunities for cooperation with TB programmes [44] there are many other disease areas where ACF in TB can work synergistically. There are several non-communicable diseases and conditions that increase the risk of developing TB like diabetes, smoking, alcohol abuse and chronic obstructive pulmonary disease. Coordination between various programmes for identifying people at risk of either disease may present opportunities for improved detection and management of several diseases [45]. Planning for bilateral coordination requires identification of focal points and formulation of shared expectations. Sharing of resources will likely decrease cost and increase impact on the detection and treatment of multiple diseases and conditions. Appropriate referral mechanisms will need to be planned. With these considerations, deliberations with technical experts, implementers of ACF and community leaders are essential.

### Step 11—*Engagement of local community and civil society*

Civil society engagement will be critical for successful implementation of ACF activities in the field. The timing of engagement of local NGOs and community groups and their specific roles and responsibilities also need to be planned in a way to ensure buy-in and acceptance. Local organizations are much more likely to know the needs, barriers and challenges faced by specific KPs that are missed by routine health services [9]. Engaging civil society in the planning of activities is a necessary step to ensure the highest levels of participation, acceptability, and impact for the people the ACF is trying to reach.

### Step 12–*Logistics*

For many ACF interventions, the workload on the laboratory for diagnostic tests and the human resources required for community awareness, screening, managing referrals, accompanying patients for diagnosis, sputum transportation, and initiation of treatment, all may rise significantly [46]. The expectations of human resources as well as diagnostics and treatment needs must be planned far in advance together with the local TB programmes to ensure a reliable supply chain. There are several examples in SEA Region for engagement with the local private sector and NGOs for implementation of ACF activities and the extra work it requires [47]. In addition, many interventions use incentives (monetary or otherwise) and decisions about the use of such approaches must be considered in the planning phase.

### Step 13—*Planning the monitoring and evaluation*

Monitoring and evaluation (M&E) will guide the ACF process, but for long term planning, ACF activities should be part of regular national and external programme reviews. As multiple ACF interventions will likely produce varied results, consolidating the lessons learnt will guide future activities. M&E activities are discussed in the next section, but the processes itself needs to be planned. A separate group of experts might need to be formed to help the planning of M&E. The collection, analysis, and dissemination of ACF results is part of the M&E and should form a continuous feedback loop for future ACF interventions.

Good planning ought to consider ACF and other alternatives for identifying people with TB, for example, by providing more service delivery points through scale-up of private sector engagement efforts, an outpost in select areas like slums, or by providing access to more rapid molecular tests. The NTP and partners should consider if these alternatives might sustainably increase the yield and reduce patient costs than repeated rounds of ACF in future [22]. A separate technical working group could be designated in the NTP to plan and monitor ACF.

## Monitoring active case finding activities

Only in the last few years have ACF activities been mainstreamed into national TB responses. Earlier, ACF was done mainly outside the NTP, or as research projects with very targeted evaluation metrics. As global and regional recommendations on ACF have broadened their acceptability and use, M&E for ACF must be incorporated into routine reporting systems. Monitoring ACF can help in improving efficiency and effectiveness not just for immediate needs of catch-up in the times of COVID-19, but also long-term strengthening of NTPs to reach the End TB targets. Monitoring ACF can help in assessing the yield against the targets, any potential additional case detection, benefits and costs to patients, and costs to the programme. Continuous feedback informs mid-course correction as well as the next iteration of ACF. The M&E can be considered in terms of process and outcome indicators which are outlined in Table 2. While the ACF is ongoing there are several indicators that assess the performance of the intervention including the acceptability and participation of people in the screening. When the outreach efforts have been completed in a given area or time, other indicators can be considered, to inform NTP planning including epidemiological and programme reviews, (for assessing the epidemiological impact if any and costs), and to provide feedback to communities where interventions are being conducted.

**Table 2:** Potential indicators for monitoring active case finding interventions.

*Process and output Indicators*		*Outcome and impact Indicators*	
*Indicator*	*Comments*	*Indicator*	*Comments*
Acceptability/Participation	If the number of eligible people is known, a basic indicator of acceptability of the ACF intervention can be constructed from calculating the number of people eligible or approached to those who agreed to be screened.	Additional TB notifications	Measures the change in TB notifications (B+/AF) in a predefined evaluation area. Additionality can be calculated using a simple pre-post analysis, TB notification trends, and employ control populations.
Number needed to screen (NNS)–E/A*	A basic indicator of the efficiency of an intervention to identify people with TB. Note that not all NNS are comparable. Doing house to house screening in remote communities with chest X-ray (CXR) will have different resource needs than a mass verbal screening event in a large urban area for instance.	Proportion of ACF yield to overall notifications	The proportion of people with TB identified by the ACF intervention among the overall TB notifications in an evaluation area provides an idea of coverage especially when comparing different demographics, key populations, by gender, age etc.
Number needed to test (NNT) E/D	Gives a general measure of how many diagnostic resources will be needed to detect someone with TB. This will change based on how loose the definition of presumptive TB is, and how sensitive the diagnostic test is.	Yield/ Additionality ratio	The ratio of cases identified by the ACF intervention to the overall change in notifications facilitates an understanding of if the intervention is reaching people who would otherwise remain undetected, or potentially if there are other reasons for an increase in notifications.
Pre-treatment loss to follow up F/E	An indicator that measures the strengths of linkages to care once people with TB are detected. ACF should not end with a diagnosis.	Early detection	There are multiple ways to measure this including patient surveys, analysis of laboratory test results (smear grading or MTB burden on Xpert) but this will involve extra data collection.
Presumptive TB rate B/A	Measures how the screening algorithm identifies people who are eligible for diagnostic testing. Using CXR can identify people without symptoms who should be tested, but also flag people with cough who are unlikely to have TB due to clear lung fields.	Costing indicators	Comparing the cost inputs of ACF to yield and/or additionality data can be done. In addition, employing patient surveys for expenditure data from the patient side.
Sputum capture rate C/B	Not all people who have presumptive TB will be able to produce a good quality sample for testing. Tools can help improve this, but as people are identified earlier in disease evolution, producing a quality sample may be more difficult.	Treatment Success G/F of those found by ACF	Part of any TB programme and can be used to examine how people found through different ACF interventions do on treatment and compare to a passive case finding cohort.
Population coverage	If overall population data is available for the intervention area, an indicator of population coverage for the ACF can be calculated.		

*The Letters (A-G) in this table correspond to the steps in the screening cascade in Fig 1.

### Process indicators for monitoring and mid-course correction

All monitoring activities for ACF interventions should strive to capture a number of data points as part of the TB screening cascade (Fig 1) including the number of people screened, those with presumptive TB based on symptoms or chest X-ray findings (screening tests), people submitting a sample for diagnostic testing, those tested by the laboratory, laboratory confirmed TB and those clinically diagnosed, and treatment initiation [48]. Table 2 describes a few process indicators and how to calculate them. A basic indicator of ACF effectiveness is the number needed to screen (NNS). This essentially provides an indication of how many people were screened to identify each person with TB. However, not all NNS is created the same, as some interventions such as home visits may be very labor intensive while others may be as simple as verbal screening in large hospital waiting rooms [49]. A similar indicator is the number needed to test, which helps understand the efficiency of diagnostic testing and the efficiency of screening for presumptive TB. Other process to monitor indicators the proportion of people with presumptive TB who provide sputum [50, 51] and are tested which can provide insight on if more attention is needed for sputum production and the strength of laboratory linkages. In addition, all ACF programmes must monitor their pre-treatment loss to follow-up [52] as detection of TB is not a sufficient endpoint and people identified through ACF should be linked to treatment.

These indicators should be part of routine monitoring for all ACF programmes and much of the data can be captured using standard forms and registers as described in Fig 1. However, there are an increasing number of mobile app technologies that can facilitate the collection of intervention level data and large investments in ACF should be accompanied by a strong data collection system [53]. India has developed a recording and reporting system for TB that is one of the best examples globally in capturing different data from the screening process [54].

In addition, intervention specific indicators may be calculated for certain types of ACF. For instance, in contact investigation, the number of people per household, or per index case may be collected [55], for mobile screening approaches, the number of camp attendees can be collected, and programmes could delineate the cascades for screening in prisons at entry, upon exit, or as a one-off process among all inmates [56]. When programmes capture the number of people screened (i.e. people given a verbal questionnaire for symptoms, or assessed with a CXR before diagnostic testing) then TB prevalence in the population screened can be calculated, and compared to national, regional or catchment area notifications [57].

TB programmes should use the data collected through the screening cascade to periodically review performance and make mid-course corrections as needed. For example, by monitoring the dropouts between people with presumptive TB and those getting tested, a project in India was able to correct the drop out issue and institute a transport system [39]. Community based organizations can assist with different steps in the cascade including promoting and monitoring the acceptability of ACF including measuring participation in the interventions (participation/eligible) and the crafting messaging that accompanies the interventions around health seeking behavior.

### Outcome indictors for national planning and decision making

The selection of outcome indicators to monitor will depend on the objectives of the ACF. To monitor the impact that ACF has on reducing the treatment coverage gap (i.e. identifying people with TB who would not have been diagnosed without the intervention) overall TB notifications should be collected for a baseline period, as well as prospectively in a larger evaluation area [20]. The yield/additionality ratio (how many TB cases the ACF produced over the absolute change in notifications) can also provide insight into how successful the ACF intervention is in reaching people who would otherwise not have been detected. A ratio close to one would indicate the intervention was reaching more people who otherwise would go without TB care, yet many people detected through ACF would eventually get diagnosed and notified through the routine systems.

ACF is expected to detect people earlier than passive case finding reaching people with undiagnosed TB who may have had mild or no symptoms and therefore, did not approach health facilities. However, how much earlier this happens is difficult to determine, and what impact early detection might have on transmission is still elusive. Studies have documented smear grading as a proxy for early/late detection [58, 59], and additional options to measure early detection include tracking and comparing CXR evolution [60] and surveys to look at health seeking behavior [58, 61]. Other recent studies have begun to document the costs associated with ACF, both from the system perspective but also out of pocket expenses for people with TB who are found through ACF [62, 63]. Although it stands to reason that people with TB who are found earlier, ideally with less severe TB, may have better treatment outcomes, certain studies that have looked at differences between actively and passively identified cohorts have not detected differences [64]. As ACF often employs community health or outreach workers, these people can be engaged to provide treatment support which can help improve outcomes. In addition, more research is needed on what the optimal periodicity of ACF could be in different populations, and its consequent impact on TB prevalence and incidence. A periodic review of ongoing ACF programmes for the outcomes can help NTPs decide if they are worth continuing.

Monitoring ACF by NTPs as a regular ongoing activity or as a part of joint monitoring missions should analyse reasons for loss using the basic TB screening cascade. Ideally the monitoring should also assess additional TB notifications, comparison of bacteriological positivity rates, and include costing analysis.

## Conclusions

The ongoing COVID-19 pandemic has revealed the fragility of the gains made by the national TB programmes and highlighted the need for active outreach to diagnose people who are missed by the routine systems. As an immediate priority during COVID-19 and post pandemic era, considerable focus will be required on ACF while keeping in mind the required efficiency because of diversion of human and financial resources towards addressing the pandemic [65]. As ACF activities are resource intensive, careful planning and monitoring are critical. We recommend that national TB programmes include careful planning for ACF in consultation with key stakeholders including the communities where ACF is to be conducted. Planning and monitoring should start with well-defined objectives for the ACF and should be rooted in national and sub-national context which considers the estimated disease burden in the population sub-group, vulnerability and size of the population, available tools and resources. Well-planned ACF that is informed by monitoring and evaluation will ensure that routine case finding activities are not compromised by diversion of financial or managerial resources. Results of ACF should be included in the epidemiological and programme reviews so that the findings can be included in updates of NSP. Implementation of ACF is in line with a rights-based and participatory approach at the community level embodied in the End-TB Strategy and Global Plan to End TB. Well-planned ACF can be a means of improving access to care for all people with TB, specifically the key populations, thereby significantly contributing to the national efforts towards ending TB.

## Abbreviations

ACF: Active case finding
KP: Key population
LTBI: Latent tuberculosis infection
NSP: National (TB) strategic plan
PLHIV: People living with HIV
SEA: South- East Asia
STP: Stop TB partnership
TB: Tuberculosis
TPT: TB preventive treatment
WHO: World Health Organization
